# Low level viraemia and the risk of virological failure in HIV infected patients

**DOI:** 10.1186/1471-2334-14-S4-O9

**Published:** 2014-05-29

**Authors:** Dragoş Florea, Ionelia Bâțan, Angelica Doana, Simona Paraschiv, Leontina Bănică, Dan Oțelea

**Affiliations:** 1 National Institute for Infectious Diseases “Prof. Dr. Matei Balş”, Bucharest, Romania

## 

Objective: to estimate the rate and the significance of low level viraemia in HIV positive patients registered in the National Institute for Infectious Diseases “Prof. Dr. Matei Balş”, Bucharest, Romania.

We retrospectively analysed the rate of HIV viral loads (VL) <200 copies/mL in patients admitted in our institute from 2011 to 2013. The patients with undetectable VLs in 2011 and low level VL in 2012 were selected and divided into three groups, according to their HIV RNA (<40, 41-50 and 51-200 copies/mL, respectively). We evaluated for each group the risk of virological failure (VL>200 and >1000 copies/mL) over a 12 months period.

A low level VL was detected in 16.2% of the 3,916 evaluated patients, the rate of HIV RNA of 51-200 copies/mL being two times higher than the rate of a VL <50 copies/mL. A number of 84 patients had a VL<200 copies/mL after being HIV RNA undetectable. In these patients the risk of virological failure over a 12 months period was not correlated with the HIV VL group or the used antiretroviral treatment (protease inhibitors PI vs. non-PI containing regimen).

A low level viraemia is a rather common event in the studied HIV patients. In previously undetectable patients a HIV RNA <200 copies/mL is not associated with an increased risk of virological failure in the next year.

